# Fine Mapping of a Novel Heading Date Gene, *TaHdm605*, in Hexaploid Wheat

**DOI:** 10.3389/fpls.2018.01059

**Published:** 2018-07-18

**Authors:** Xueying Zhang, Guoxiang Liu, Lichao Zhang, Chuan Xia, Tianxiang Zhao, Jizeng Jia, Xu Liu, Xiuying Kong

**Affiliations:** Key Laboratory of Crop Germplasm Resources and Utilization, Ministry of Agriculture, National Key Facility for Crop Gene Resources and Genetic Improvement, Institute of Crop Sciences, Chinese Academy of Agricultural Sciences, Beijing, China

**Keywords:** wheat, EMS mutant, heading date gene, fine mapping, physical map

## Abstract

The heading date is critical in determining the adaptability of plants to specific natural environments. Molecular characterization of the wheat genes that regulate heading not only enhances our understanding of the mechanisms underlying wheat heading regulation but also benefits wheat breeding programs by improving heading phenotypes. In this study, we characterized a late heading date mutant, *m605*, obtained by ethyl methanesulfonate (EMS) mutation. Compared with its wild-type parent, YZ4110, *m605* was at least 7 days late in heading when sown in autumn. This late heading trait was controlled by a single recessive gene named *TaHdm605*. Genetic mapping located the *TaHdm605* locus between the molecular markers cfd152 and barc42 on chromosome 3DL using publicly available markers and then further mapped this locus to a 1.86 Mb physical genomic region containing 26 predicted genes. This fine genetic and physical mapping will be helpful for the future map-based cloning of *TaHdm605* and for breeders seeking to engineer changes in the wheat heading date trait.

## Introduction

Heading date (flowering time) in crops is associated with the timing of the floral transition and is one of the most important agronomic traits that determines the distribution and regional adaptability of plants, thereby affecting crop production. Plants have evolved multiple genetic pathways that integrate both internal signals and extrinsic stimuli to adapt flowering time to different environmental conditions. Isolation and functional study of heading related genes will provide better understanding of the genetic pathways that control the flowering time of plants and could offer effective strategies for engineering high-yield varieties that can adapt to different climate conditions and changing environments (Jung and Muller, 2009).

Wheat (*Triticum aestivum* L, 2*n* = 6*x* = 42, AABBDD) is grown in a wide range of environments all over the world. Suitable heading date is an important determinant that contributes to precise adjustment for seasonal changes, which is important to maximize crop yield. Many studies have been performed to clarify the genetic control of heading date in wheat and have identified the vernalization requirement and photoperiod sensitivity as two main components in the regulation of heading date in wheat.

Vernalization is the requirement for a plant to undergo cold temperatures before flowering. The wheat vernalization requirement is mainly controlled by the *VRN1*, *VRN2*, *VRN3*, and *VRN4* genes, and the characterization of these four genes has advanced our understanding of vernalization regulatory pathways (Yan et al., 2004, 2006; Fu et al., 2005; Li and Dubcovsky, 2008; Yoshida et al., 2010; Kippes et al., 2014). *VRN1*, isolated in diploid wheat using a map-based cloning method, has been shown to share high sequence similarity with the *Arabidopsis* MADS-box gene *AP1/FRUITFULL*. The central role of *VRN1* in the leaves is to maintain low transcript levels of the flowering repressor gene *VRN2* after vernalization and to ultimately regulate timely flowering in the spring (Chen and Dubcovsky, 2012). The *VRN2* gene encodes a zinc finger-CCT domain transcription factor and acts as a dominant flowering repressor that can be down-regulated by vernalization (Yan et al., 2004). It has been postulated that the VRN2 protein suppresses the expression of the flowering-integrator gene *FT* (*VRN3*), which is a mobile signaling protein that moves from the leaves to the shoot apical meristem to induce flowering. Transgenic analysis in winter wheat indicated that reduced *VRN2* expression leads to increased transcription of *VRN1* and accelerated flowering (Yan et al., 2004; Dubcovsky et al., 2006; Li et al., 2011; Chen and Dubcovsky, 2012). Further studies have also demonstrated that *VRN3* regulates *VRN1* transcription through interactions with FDL2. Most importantly, *VRN3* is an integrator of vernalization and photoperiod pathways (Yan et al., 2006; Trevaskis et al., 2007; Li and Dubcovsky, 2008). For these three vernalization genes, the combination of the recessive *vrn-1* allele, dominant *Vrn-2* allele and recessive *vrn-3* allele is associated with winter growth habits, and a mutation in any one or more of these three genes results in a variant with a spring growth habit (Tranquilli and Dubcovsky, 2000; Yan et al., 2006). *VRN4* has been found only in the D genome *(VRN-D4*). The detection of significant interactions between *VRN-D4* and other four genes controlling vernalization requirement (*Vrn-A1*, *Vrn-B1*, *Vrn-D1*, and *Vrn-B3*) confirmed that *VRN-D4* acts as part of the vernalization pathway and that it is either upstream of or part of the regulatory feedback loop involving the *VRN1*, *VRN2*, and *VRN3* genes. Subsequent studies have demonstrated that *VRN-D4* originated from the insertion of a ∼290 kb region carrying a copy of *VRN-A1* from chromosome arm 5AL into the proximal region of chromosome arm 5DS (Kippes et al., 2015).

The photoperiod response in wheat is primarily determined by the three dominant genes *Ppd-A1*, *Ppd-B1*, and *Ppd-D1*, which belong to three homologous loci on chromosomes 2A, 2B, and 2D, respectively. Wheat is a LD plant, and SD conditions can delay its heading time. The *Ppd1* gene acts as a regulator that reduces the delay in heading time under SD conditions (Murai et al., 2003). Recent studies have suggested that diverse alleles and epigenetic modification of the *Ppd1* genes may contribute to photoperiod sensitivity and affect the heading date in wheat (Distelfeld et al., 2009; Dhillon et al., 2010; Guo et al., 2010; Diaz et al., 2012; Sun et al., 2014). Another flowering pathway called the *GI*-*CO*-*FT* regulation pathway, which was found in both *Arabidopsis* and rice, has also been identified in the wheat flowering process. Studies have already demonstrated that *VRN3* is the wheat *FT* gene (Yan et al., 2006), and its upstream genes *TaGI1* and *TaCO* were cloned in wheat using the homology cloning method (Nemoto et al., 2003; Zhao et al., 2005).

When wheat cultivars were exposed to an optimal photoperiod and vernalization treatments, there were still differences in flowering time among the wheat accessions, which is usually referred to as earliness *per se* (*Eps*) (Slafer, 1996). The *Eps-Am1* locus was identified from the *Triticum monococcum* chromosome 1A^m^ (Bullrich et al., 2002). However, it exhibited a large effect on the heading date and significant epistatic interactions with the photoperiod and vernalization treatments; *Eps-Am1* also exhibited a significant interaction with temperature. Moreover, *Eps-Am1* affected spike development and spikelet number (Bullrich et al., 2002; Faricelli et al., 2010). Further study identified *EARLY FLOWERING 3* (*ELF3*) as a candidate gene (Alvarez et al., 2016).

Although these studies provide a basic understanding of the control of the flowering process in wheat, the regulatory networks of flowering in wheat and their underlying molecular mechanisms are not well understood. To solve these problems, more wheat heading date genes need to be discovered. In the present study, we report the identification of a novel heading date gene *TaHdm605* and illustrate fine mapping of the heading date gene in bread wheat; these results could serve as a framework for map-based cloning and for marker-assisted selection in wheat breeding programs.

## Materials and Methods

### Plant Materials

The *m605* mutant originated from the seeds of the elite wheat variety YZ4110 (*vrn-A1*, *vrn-B1*, *Vrn-D1*, *vrn-2*, *vrn-3*, *Ppd-A1b*, *Ppd-B1b*, and *Ppd-D1a*), which was treated with EMS (Zhao et al., 2009). The F_2:3_ populations derived from *m605* crossed with an early heading variety, Chun47 (*vrn-A1*, *vrn-B1*, *Vrn-D1*, *Vrn-2*, *vrn-3*, *Ppd-A1b*, *Ppd-B1b*, and *Ppd-D1a*) and wild-type YZ4110, respectively, were used for genetic analysis. The genotypes of the *Vrn* and *Ppd* loci of WT YZ4110, the *m605* mutant and Chun47 were detected by the primers listed in **Supplementary Table S1** (Fu et al., 2005; Yan et al., 2006; Zhu et al., 2011; Nishida et al., 2013). The F_2_ population derived from the *m605* mutant crossed with wild-type YZ4110 was used to test the segregation ratio of the heading date phenotype. The F_2_ population from the cross of Chun47 × *m605* was used to perform fine mapping. Fifteen seeds from the F_2_ segregating population as well as the parents were planted in 2-m rows spaced 30 cm apart in fields in Beijing (N39°49’, E 116°39’) and Xinxiang (N35°18’, E113°55’), China, between 2010 and 2013. To ensure the accuracy of the results, the phenotypes of the F_2_ plants were further validated by using the F_3_ families. F_3_ families containing 25 seeds from every F_2_ plants were planted in rows for verifying the phenotypes of the F_2_ plants in fields in Beijing and Xinxiang between 2011 and 2014. To avoid the damage caused by cold weather during hard winters, the seedlings planted in Beijing were covered with plastic film. For main shoot apex development analysis, the mutant plants of *m605* and YZ4110 were grown in greenhouse with vernalized in 4°C for 3 weeks. The room temperature at the greenhouse was maintained at 22°C during the 16-h light period and at 18°C during the 8-h dark period, and the average relative humidity was 75%. The floral primordium samples of *m605* and YZ4110 were collected between 9 and 10 AM after 8 and 10 weeks, respectively.

### Data Analysis

For the comparison of days to heading and other agronomical traits between YZ4110 and *m605*, the data of agronomical traits from 20 plants were tested and recorded from 2009 to 2016. One-way analysis of variance was performed in Excel. The F_2_ population from the cross of Chun47 × *m605*, plant height, spike length, effective tiller number, grain number per spike, and 1,000-kernel weight of each individual progeny were recorded. The data analysis was conducted using SPSS software. ^∗^Indicates 0.01 < *p* < 0.05, ^∗∗^indicates *p* < 0.01.

### DNA Extraction and Polymerase Chain Reaction (PCR)

Total genomic DNA from the Chun47 × *m605* F_2_ population was isolated from leaves by using a cetyltrimethylammonium bromide (CTAB) method (Saghai-Maroof et al., 1984). PCR was performed in a Veriti thermal cycler (Life Technologies, United States) in 10 μl reactions containing 25 ng of each primer, 30 ng of genomic DNA, and 5 μL of 2 × PCR MasterMix (Tiangen, Beijing, China). The PCR conditions were as follows: denaturation at 94°C for 10 min, followed by 40 cycles of 94°C for 45 s, 55°C for 45 s, and 72°C for 45 s and a final extension for 10 min at 72°C. PCR products were mixed with 2 μl of loading buffer (98% formamide, 10 mM EDTA, 0.25% bromophenol blue, and 0.25% xylene cyanol), separated on 5% denaturing polyacrylamide gels and visualized using silver staining. Silver staining was performed as follows: rinse with water for 10 s, silver stain (1 g/L AgNO_3_) for 8 min, rinse with water for 10 s, develop for 5–10 min (16 g/L NaOH, 0.75% HCHO), fix for 1 min (7.5 g/L Na_2_CO_3_), and rinse with water for 5 min.

### Molecular Marker Analysis and Polymorphic Marker Development

F_2_ plants from the cross of Chun47 and *m605* were used to establish early and late bucks for bulked segregant analysis (BSA). The DNAs of the 10 homozygous plants that exhibited early heading dates and late heading dates, based on a validation of the heading date of the F_3_ family, were pooled in equal quantities to construct an early bulk sample and a late bulk sample, separately. Wheat genomic simple sequence repeat (SSR) markers from the GrainGenes website^1^, with an average distance of 10 cM between adjacent markers, were used for screening the polymorphisms between the two parents and two bulk samples. The resulting polymorphic markers were used to genotype the F_2_ populations.

The marker sequences linked to *TaHdm605* were used to perform BLAST searches of the *Aegilops tauschii* genome sequence (Jia et al., 2013) to develop new polymorphic markers. The obtained sequences were used as templates to design primers with Primer3^2^ with the following parameters: amplification product size of 120–600 bp, primer length of 17–24 bp, Tm of 55–65°C with the optimum at 60°C, and GC content of 40–60%. The designed primers were first screened for polymorphisms between the parental lines, as well as the early and late DNA bulks. The chromosome 3DL-specific polymorphic markers were used for genotyping the F_2_ populations to construct a high-density genetic linkage map.

### High-Density Genetic Linkage Map Construction

Chi-squared (χ^2^) tests for goodness-of-fit were used to evaluate the deviations of the observed data from the theoretically expected segregation ratios. A linkage map with the genetic distance between the markers and the *TaHdm605* gene was determined using Mapmaker 3.0, with a logarithm of odds (LOD) score threshold of 3.0 (Lincoln et al., 1992). A genetic map was constructed with the software Mapdraw V2.1 (Liu and Meng, 2003).

### Candidate Gene Analysis and Comparative Genomics Analysis of the TaHdm605 Locus Among Different Species

The nearest marker sequences flanking the *TaHdm605* gene were used to perform BLAST searches of the wheat chromosome 3DL survey sequences (International Wheat Genome Sequencing Consortium (IWGSC) RefSeq v1.0). Gene annotation for the obtained genomic sequence surrounding the *TaHdm605* gene was conducted by using the TriAnnot pipeline^3^ and BLAST analysis tools from NCBI^4^. The genes expression pattern was determined by blasting the predicted CDS sequences to WheatEXP^5^ with an e-value cutoff of 1e-10. The identified gene sequences were used as query sequences to BLAST search the *Brachypodium*^6^, rice^7^ and sorghum^8^ genome sequences with an *e*-value cutoff of 1e-10. The orthologous gene pairs among the different species were compared within the homologous genomic regions. The inversion region of the *TaHdm605* locus was identified by projecting the genetic markers of *A. tauschii* onto the *Brachypodium* and rice chromosomes (Luo et al., 2013).

## Results

### Phenotype of the *m605* Mutant

**FIGURE 1:**
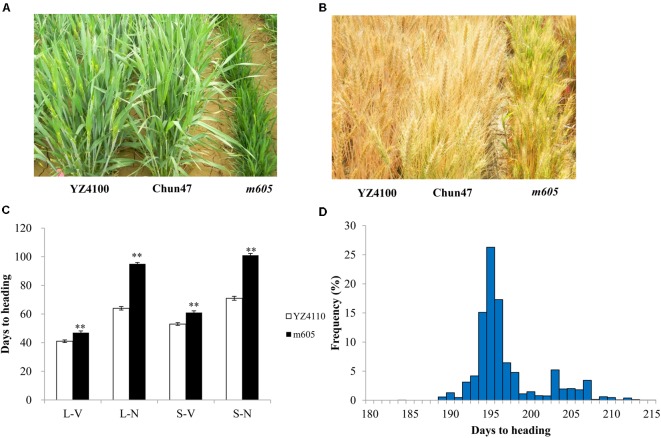
Phenotypes of wild-type YZ4110, late heading mutant *m605* and early heading parent Chun47 as planted in the field and in controlled conditions. **(A)** The phenotypes of YZ4110, Chun47, and *m605* at the heading stage when planted in the field. **(B)** The phenotypes of YZ4110, Chun47, and *m605* at the ripening stage when planted in the field. **(C)** Days to heading of YZ4110 and *m605* under controlled conditions. L-V, long day and vernalized; L-N, long day and non-vernalized; S-V, short day and vernalized; S-N, short day and non-vernalized. The data in **(C)** represent the means of three replicates, and the error bars indicate the standard deviation. ^∗^ and ^∗∗^ represents 0.01 < *p* < 0.05 and *p* < 0.01, respectively, based on a Student’s *t*-test. **(D)** Frequency distribution of heading date in the F_2_ population derived from Chun47 × *m605* planted in Xinxiang, Henan province.

The wheat heading date mutant *m605* was obtained by mutagenesis of YZ4110 seeds with EMS (**Figures 1A,B**). To measure the difference in heading date between the *m605* mutant and YZ4110, we investigated the days to heading of *m605* and YZ4110 in 10 environments. The results show that the average delay in the heading date of *m605* was approximately 12.1 days, and the difference between *m605* and YZ4110 was significant (**Supplementary Table S2**). Furthermore, the *m605* mutant was characterized by a clustered growth habit accompanied by other characteristics, such as dwarfing and short spike length (**Table 1**). We also performed main shoot apex development analysis of the *m605* and YZ4110, as shown in **Supplementary Figure S1**, and the development of floral primordium in the *m605* was apparently slower than that in YZ4110. Moreover, we compared the differences in heading date between *m605* and YZ4110 under controlled conditions. As shown in **Figure 1C**, and the three-way ANOVA analysis (**Supplementary Table S3**), in both LD and SD conditions, the differences in heading date between *m605* and YZ4110 under non-vernalized conditions were significantly greater than those under vernalized conditions (*p* < 0.0001), whereas the performance of *m605* and YZ4110 under LD and SD conditions was not significantly different (*p* = 0.5172). These results indicated that *TaHdm605* interacted with vernalization treatments but did not follow the photoperiod pathway, implying that temperature may significantly influence the function of the *TaHdm605* gene.

**Table 1:** The comparison of agronomically important traits in YZ4110 and *m605*.

Accession	Years	Location	No.^#^	Days to heading	Plant height	Spike length	Effective tiller number	Spikelet number	Grain number per spike	1,000-kernels weight
YZ4110	2012	Beijing	20	234.25 ± 0.85	70.2 ± 0.58	10.1 ± 0.33	16.6 ± 1.50	22.4 ± 0.4	49.6 ± 1.63	44.81 ± 1.39
*m605*			20	245.15 ± 0.67^∗∗^	54.3 ± 0.99^∗∗^	8.3 ± 0.25^∗∗^	15 ± 1.34	13.4 ± 0.4^∗∗^	18.4 ± 0.74^∗∗^	28.43 ± 1.63^∗∗^
YZ4110	2014	Beijing	20	219.1 ± 0.71	70.08 ± 0.68	10 ± 0.21	7.17 ± 0.32	21 ± 0.47	52 ± 2.26	52.80 ± 1.51
*m605*			20	239 ± 0.92^∗∗^	53 ± 0.83^∗∗^	7.67 ± 0.25^∗∗^	8.17 ± 1.42	13.83 ± 0.40^∗∗^	22.83 ± 1.92^∗∗^	37.82 ± 4.75^∗^
YZ4110	2015	Beijing	20	260.15 ± 0.59	75.4 ± 1.65	10.3 ± 0.22	10.8 ± 1.31	22.8 ± 0.45	43.4 ± 1.33	38.33 ± 1.84
*m605*			20	265.85 ± 0.88^∗∗^	44.5 ± 0.76^∗∗^	6.4 ± 0.33^∗∗^	7 ± 1.18	9.2 ± 0.8^∗∗^	13.4 ± 1.47^∗∗^	24.32 ± 0.36^∗∗^
YZ4110	2016	Beijing	20	224.2 ± 0.61	69.3 ± 0.83	9.3 ± 0.68	12.4 ± 2.4	20.8 ± 1.02	38.8 ± 3.88	45.74 ± 2.26
*m605*			20	235.05 ± 0.76^∗∗^	51.8 ± 2.13^∗∗^	6.4 ± 0.4^∗∗^	11.8 ± 0.73	11.6 ± 0.75^∗∗^	19.6 ± 2.04^∗∗^	30.71 ± 1.88^∗∗^

*^#^Indicates the number of plants analyzed. ^∗^p < 0.05; ^∗∗^p < 0.01.*

### Genetic Analysis of the TaHdm605 Gene in Hexaploid Wheat

To test the segregation ratio of the heading date phenotype, the F_2_ populations derived from the *m605* mutant crossed with the wheat cultivar Chun47 were planted in Beijing and Xinxiang (Henan province), respectively. A total of 2,480 F_2_ plants contained 587 late and 1,893 early heading plants in Xinxiang, in Henan province, and these plants fit a 1:3 ratio (χ^2^ = 2.34, *p* > 0.05) (**Figure 1D**); 746 F_2_ plants contained 205 late and 541 early heading plants in Beijing, and these plants also fit a 1:3 ratio (χ^2^ = 2.45, *p* > 0.05). The agronomic data from the Chun47 × *m605* F_2_ population was listed in **Supplementary Table S4**. We also tested the segregation ratio of the heading date phenotype by using the F_2_ population derived from the *m605* mutant crossed with wild-type YZ4110. In the 226 F_2_ plants planted in Beijing, there were 54 late and 172 early plants, as expected of a 1:3 ratio (χ^2^ = 0.15, *p* > 0.05). These results suggested that a single recessive gene, temporarily named *TaHdm605*, controlled the late heading phenotype in the *m605* mutant. Additionally, through analyzing the agronomic traits of the F_2_ population, we found that the heading date of *m605* had significantly negative correlations with 1,000-kernel weight, plant weight, grain number per plant, grain number per spike, spikelet number, spike length, and plant height (**Supplementary Table S5**).

### Initial Mapping of the Heading Date Gene *TaHdm605* by Using Public Markers

**FIGURE 2:**
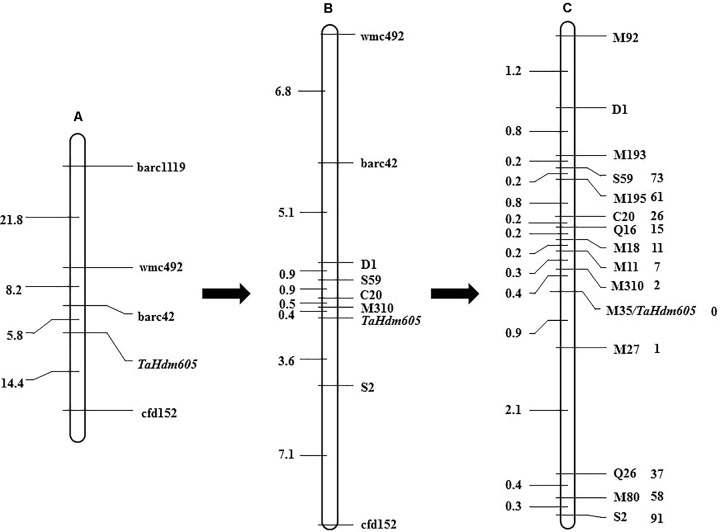
Genetic map of the heading date gene *TaHdm605*. **(A)** Initial mapping of the heading date gene *TaHdm605* obtained with public markers and 120 F_2_ plants. **(B)** Genetic mapping of the heading date gene *TaHdm605* obtained with markers developed by using the *A. tauschii* genome sequence (Ver. 0.1). **(C)** High-resolution genetic map of *TaHdm605.* The genetic distances in cM are shown on the left, and the markers are shown on the right. The numbers on the right of the markers represent the recombinants of the corresponding markers.

To map the *TaHdm605* gene, 871 publicly available SSR markers distributed on the 21 wheat chromosomes, with an average distance of 10 cM, were used to screen the polymorphisms between the two parents as well as between early and late DNA bulk samples. As a result, four chromosome 3D specific markers, barc1119, wmc492, barc42, and cfd152, were found to have polymorphisms between both parents and bulk samples. Then, a genetic linkage map of *TaHdm605* was constructed with the four linked marker loci by using 120 F_2_ plants from the cross between *m605* and Chun47. Lastly, *TaHdm605* was initially mapped between the markers barc42 and cfd152, with genetic distances of 5.8 and 14.4 cM, respectively (**Figure 2A**).

### Fine Genetic Mapping of *TaHdm605* by Using the *A. tauschii* Genome Reference Sequence

To obtain markers more closely linked to the *TaHdm605* gene, the sequences of the markers barc42 and cfd152 were used to perform a BLAST search against the *A. tauschii* genome sequence (Ver. 0.1) to develop additional markers (Jia et al., 2013). As a result, a 63 Mb genomic region containing the *TaHdm605* locus was identified, and a total of 1,015 new SSR markers were developed. Among these markers, five polymorphic markers, C20, D1, S2, S59, and M310, were screened and deployed to genotype the F_2_ segregating population of 3,226 plants. The results showed that *TaHdm605* was located between markers M310 and S2 with genetic distances of 0.4 and 3.6 cM (**Figure 2B**). Later in this study, a new version of the *A. tauschii* genome sequence (Ver. 1.1) with higher quality became available (our unpublished data). By using this new sequence information, nine more markers, Q16, Q26, M11, M18, M27, M35, M80, M92, M193, and M195, were developed and used to screen the recombinants (**Supplementary Table S6**). Finally, *TaHdm605* was mapped between the markers *M310* and *M27* with genetic distances of 0.4 and 0.9 cM and was found to co-segregate with the marker *M35* (**Figure 2C**). Additionally, we checked the reported QTLs located on chromosome 3D, revealing only one QTL, *QEet.fal-3DL*, reported for heading date (Paillard et al., 2004). Further analysis indicated that *QEet.fal-3DL* is located between the markers Xgwm645 and Xgwm383a. By blasting these two markers sequence to the wheat genome reference sequence (CS; IWGSC RefSeq v1.0), we found that this QTL was not located in our candidate region. These results suggested that *TaHdm605* is a novel gene for heading date.

### Candidate Gene Analysis by Using Wheat Genome Reference Sequences

**FIGURE 3:**
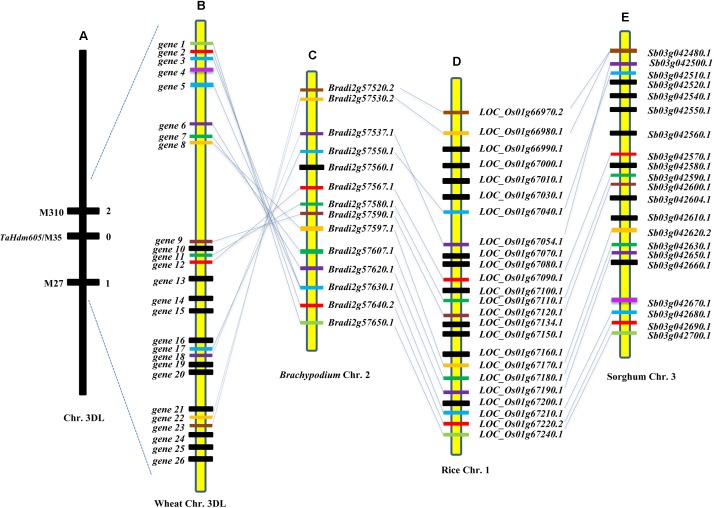
Fine mapping of *TaHdm605* and comparative genomics analysis of the surrounding region in wheat, *Brachypodium*, rice, and sorghum. **(A)** Genetic linkage map of the heading date gene *TaHdm605* on wheat chromosome arm 3DL. Horizontal lines indicate mapping marker locations, and the number of recombinants is shown on the right. **(B)** Physical map of the *TaHdm605* locus based on Chinese Spring 3DL sequences. The horizontal lines represent the predicted wheat genes. **(C)** Orthologous genomic region of *TaHdm605* on *Brachypodium* chromosome 2. **(D)** Orthologous genomic region of *TaHdm605* on rice chromosome 1. **(E)** Orthologous genomic region of *TaHdm605* on sorghum chromosome 3. For **(B–E)** orthologs are indicated by diagonal lines.

The reference sequence of the bread wheat Chinese Spring (CS; IWGSC RefSeq v1.0) was released during the process of our mapping work on *TaHdm605*. To support future identification of the candidate gene of *TaHdm605*, the sequences of M27 and M310 were used as query sequences to perform BLAST searches of the CS 3DL reference sequence. As a result, a contiguous sequence covering the markers M27 and M310 was identified. The physical interval between the two markers was 1.86 Mb and contained 26 predicted genes (**Figures 3A,B**). To obtain the functional information for these 26 predicted genes, we first conducted functional annotation for these genes (**Supplementary Table S7**). For these 26 predicted genes, seven genes (*gene8*, *gene11*, *gene13*, *gene15*, *gene16*, *gene22*, and *gene25*) were annotated only as expressed proteins with unknown function. The proteins encoded by the remaining 19 genes had a variety of functions. However, none of these 26 predicted genes was a previously reported gene that related to regulation of heading date or flowering time in wheat or any other species. Moreover, we checked the expression patterns of these 26 genes in different tissues by using the sequences of the 26 predicted genes to blast WheatEXP (see footnote 5). As a result, 11 genes were expressed in all of the five test tissues at different levels, four genes were expressed in only one or some of the five test tissues, and the remaining 11 genes were not Blast the hits with high similarity on chromosome 3D (**Supplementary Figure S2**).

### Comparative Genomics Analysis of the T*aHdm605* Region Among Wheat, *Brachypodium*, Rice, and Sorghum

We also assessed the collinearity of the syntenic genomic region around the *TaHdm605* locus in wheat compared to *Brachypodium*, rice, and sorghum. First, collinear regions spanning 150 kb, 123.5 kb, and 148 kb were identified in *Brachypodium*, rice, and sorghum, respectively (**Figures 3C–E**). Detailed analysis revealed a 150 kb segment in *Brachypodium* chromosome 2 containing 14 predicted genes (*Bradig2g57520-Bradig2g57650*), a 123.5 kb segment in rice chromosome 1 containing 24 predicted genes (*Os01g66970–Os01g67240*) and a 148 kb segment in sorghum chromosome 3 containing 21 predicted genes (*Sb03g042480–Sb03g042700*) (**Figures 3B–E**). Then, the sequences of the 26 predicted wheat genes were used to perform BLAST searches of the genome sequences of *Brachypodium*, rice, and sorghum to evaluate the collinearity of wheat with the other three species around the *TaHdm605* locus. On the whole, these comparative analyses suggested that out of the 26 predicted CS genes, 14 are orthologous to genes in *Brachypodium* and rice and 15 are orthologous to genes in sorghum. Of the 14 predicted *Brachypodium* genes, 13 are orthologs of rice genes and 14 are orthologs of sorghum genes. Of the 24 predicted rice genes, 13 are orthologs of sorghum genes (**Supplementary Table S7**). These results implied that the genomic region around the *TaHdm605* locus in wheat did not share a high degree of collinearity with the corresponding regions in the other three genomes.

**FIGURE 4:**
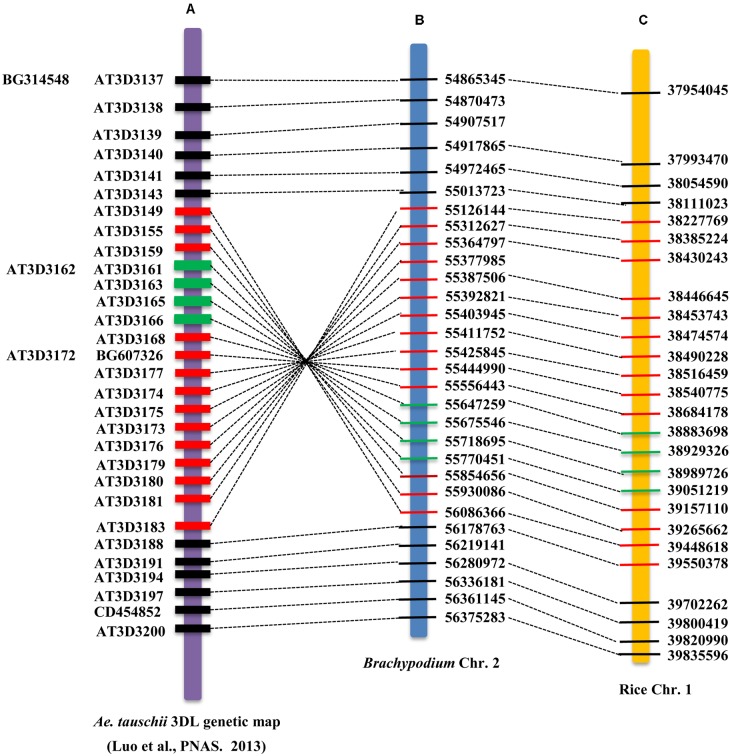
Schematic of the border and position of the genomic inversion around the *TaHdm605* locus. The dashed lines indicate the collinear positions of the markers from *A. tauschii* on *Brachypodium* chromosome 2 and rice chromosome 1. The red rectangles represent the region of the genomic inversion in *A. tauschii*, *Brachypodium* and rice. The green rectangles indicate the region of the *TaHdm605* locus covered by the markers *M27* and *M310*. **(A)** High-resolution genetic map of *TaHdm605* on *A. tauschii*, with single nucleotide polymorphism (SNP) markers shown on the left. **(B,C)** Physical maps of *TaHdm605* on *Brachypodium* chromosome 2 and rice chromosome 1, with physical position on the right.

Moreover, when projecting the high-resolution genetic map of *A. tauschii* onto the *Brachypodium* and rice chromosomes, we found that the region around the *TaHdm605* locus is part of an ∼15.9 Mb inversion in the *A. tauschii* genome between the markers AT3D3149 and AT3D3183, and the inversion region in the wheat 3D chromosome is also approximately 15.9 Mb in length (CS; IWGSC RefSeq v1.0). The corresponding regions in *Brachypodium* and rice were approximately 0.96 Mb and 1.17 Mb, respectively (**Figure 4**).

## Discussion

Wheat is one of the world’s most important staple food crops. Heading date (flowering time) is an important agronomic trait that not only determines the distribution and regional adaptability of a crop but also influences its yield (Jung and Muller, 2009). Taking the heading date regulatory gene *Ppd1* and rice *Ghd7* as examples, these two genes also directly regulate crop growth and yield (Xue et al., 2008; Boden et al., 2015). Therefore, identifying genes that regulate heading is beneficial for crop improvement including appropriate heading date and high yield in the process of modern plant breeding.

Plants have developed multiple mechanisms to ensure that heading or flowering occurs at the correct time in a given environment, which were regulated by a series of genes. In wheat, *VRN1*, *VRN2*, *VRN3*, and *VRN-D4* in the vernalization pathway and *Ppd-1A*, *Ppd-1B*, and *Ppd-1D* in the photoperiod pathway have been cloned and functionally studied (Zheng et al., 2013; Kippes et al., 2015), however, very little is known about the other genes that participate in these two pathways or about genes active in other heading date regulatory pathways. Therefore, characterization of heading date mutants and mapping new heading date regulatory genes are of great significance for clarifying the mechanisms regulating wheat heading date. In this work, we characterized a late heading mutant, *m605*, which was obtained by EMS mutagenesis of YZ4110 seeds. Our results indicate that the mutant *m605* exhibited about a 12.1-day delay in heading date compared to YZ4110. In china, the light and temperature conditions vary greatly between different ecological regions, therefore, a delay in the heading date of *m605* may be beneficial in some environments. As a mutant, some undesirable changes in several yield components were detected in mutant *m605*, which will limit its direct and wide use in future breeding research. However, after we clone the causative gene of mutant *m605*, we will determine its sequence polymorphisms in wheat varieties distributed in different ecological conditions, which allows us to detect the natural variant that could affect wheat growth habits and heading dates. Ultimately, we will select the suitable natural alleles of a specific wheat variety in the breeding programs. Moreover, studies conducted on this gene will also enrich our knowledge and understanding of the regulation of flowering time in wheat.

Genetic analysis suggested that the late heading in the *m605* mutant is controlled by a single recessive allele of the *TaHdm605* gene. By use of publicly available markers, the late heading gene *TaHdm605* was mapped to a region of chromosome 3DL spanned by markers barc42 and cfd152 with genetic distances of 5.8 cM and 14.4 cM, respectively. Constructing the high-resolution genetic linkage map and the physical map containing the candidate gene is among the most important steps toward map-based cloning of the target gene. At the beginning of the mapping of the *TaHdm605* gene, the assembled reference of the sequence of hexaploid wheat was not available. However, the genome sequence of the wheat D-genome progenitor *A. tauschii* was identified during our study (Jia et al., 2013). The availability of the genome sequence resource was of great utility in further marker development for mapping the *TaHdm605* gene. Fortunately, the initial mapping using public markers already located the *TaHdm605* on the 3DL chromosome, which meant that using the genome sequence of *A. tauschii* was a feasible and efficient way to develop new markers linked to *TaHdm605*. With this strategy, 15 polymorphic markers linked to *TaHdm605* were successfully developed, and the *TaHdm605* gene was mapped to a 1.3 cM interval. To date, none of the heading date regulatory genes or QTLs have been reported in this region, indicating that *TaHdm605* is a novel gene related to heading date in wheat.

Due to the great advances in sequencing technologies and assembly strategies, wheat genome sequencing has achieved very remarkable progress, and the new version of the chromosome-based reference sequences with the whole genome assembly and the emmer wheat genome sequence have recently become available (International Wheat Genome Sequencing Consortium [IWGSC], 2014; Avni et al., 2017); these advances have provided valuable sequence information for the cloning of important genes in the future. Later in the study, with the release of the wheat genome assembly (IWGSC RefSeq v1.0), additional sequences could be used to construct the physical map around the *TaHdm605* gene. By using this sequence information, we located *TaHdm605* in a 1.86 Mb genome region that contained 26 predicted genes. Based on the sequence analysis and functional annotation of these 26 genes, none of the genes were previously reported genes that function in regulating heading date or flowering time in plants.

In the future work, to clone the candidate gene of *TaHdm605*, we must sequence these 26 genes in YZ4110 and *m605* and find the mutated nucleotides in *m605*. In the meantime, one thing we cannot ignore is that some causative mutant genes may be located in non-coding regions, such as promoter or intergenic regions. If we do not determine the causative mutant site in gene coding regions for *m605*, different approaches may be required to detect the causative mutation in the non-coding region or promoter region. Once we obtain the causative mutation, the candidate gene of *TaHdm605* can be determined based on the composition of the mutated nucleotide sites in the recombinants. Therefore, the results collected here will serve as a valuable framework for future map-based cloning of *TaHdm605* and for breeders to engineer changes in this trait.

## Author Contributions

XK, JJ, and XL designed the research. GL and TZ collected the plant materials and constructed the populations. GL and XZ performed the experiments. GL, LZ, XZ, CX, JJ, and XK analyzed the data. LZ and XK wrote the paper.

## Conflict of Interest Statement

The authors declare that the research was conducted in the absence of any commercial or financial relationships that could be construed as a potential conflict of interest.

## Footnotes

1 http://wheat.pw.usda.gov/GG3

2 http://bioinfo.ut.ee/primer3-0.4.0/

3 https://urgi.versailles.inra.fr/triannot/?pipeline

4 https://www.ncbi.nlm.nih.gov

5 https://wheat.pw.usda.gov/WheatExp/

6 http://mips.helmholtz-muenchen.de/plant/brachypodium/

7 http://rice.plantbiology.msu.edu/

8 http://mips.helmholtz-muenchen.de/plant/sorghum/

## Supplementary Material

FIGURE S1 Comparision of floral primordium betweenYZ4110 and *m605*. **(A,C)** Floral primordium of YZ4110. **(B,D)** Floral primordium of *m605.*

FIGURE S2 The expression profiles of the candidate genes on WheatEXP.

TABLE S1 Details for primers used in the phenotype detection.

TABLE S2 The comparison of days to heading between YZ4110 and *m605.*

TABLE S3 The significance test of the interactions between *TaHdm605* alleles, photoperiods, and vernalization treatments.

TABLE S4 Agronomic trait datas of the F_2_ population in the crossing of Chun47 × *m605* in Beijing and Xinxiang.

TABLE S5 Agronomic trait analysis based on the F_2_ population from the cross Chun47 × *m605*.

TABLE S6 Newly developed polymorphic SSR markers tightly linked to the *TaHdm605* locus.

TABLE S7 Gene annotation of the *TaHdm605* genomic region on the Chinese Spring chromosome 3DL.

## Abbreviations

EMS: ethyl methanesulfonate
LD: long-day
SD: short-day.
