# Supplementary figures and images for: Fine Mapping of a Novel Heading Date Gene, TaHdm605, in Hexaploid Wheat
Source: Front Plant Sci. 2018 Jul 18;9:1059. doi: 10.3389/fpls.2018.01059 (PMC6058285; doi:10.3389/fpls.2018.01059)

Figure S1

(A)

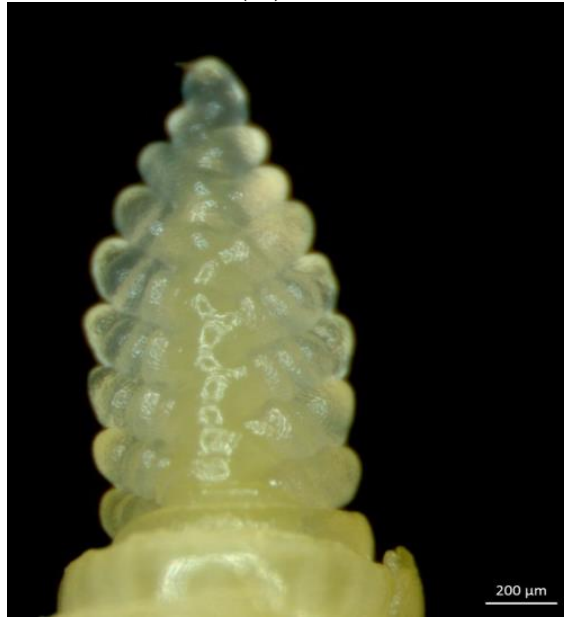

(B)

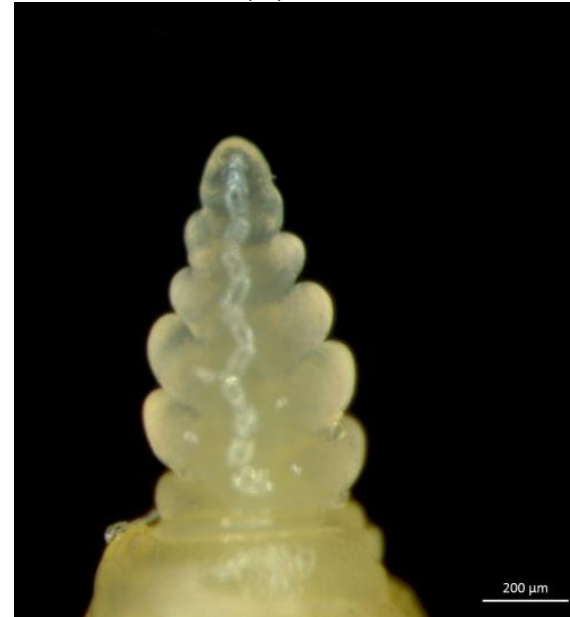

(C)

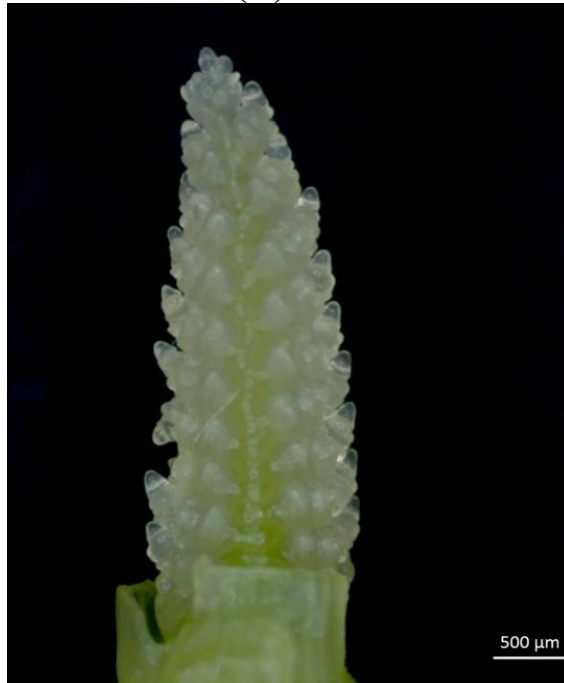

(D)

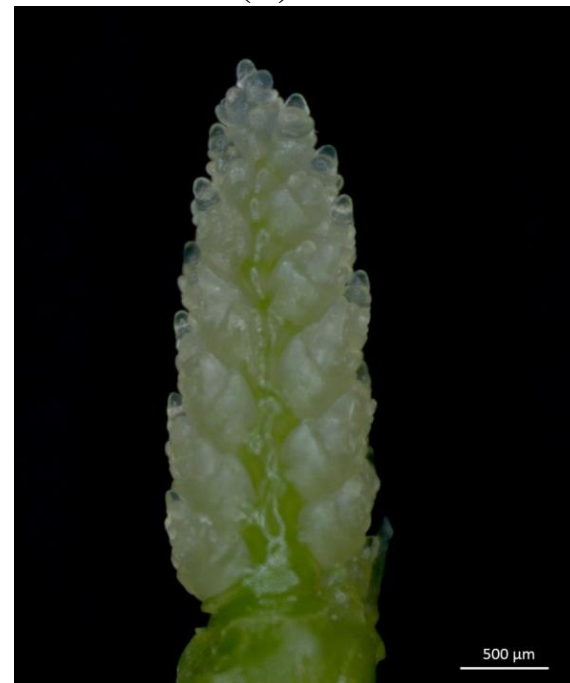

Supplement: FIGURE S1 — Comparision of floral primordium betweenYZ4110 and m605. (A,C) Floral primordium of YZ4110. (B,D) Floral primordium of m605. [file Image_1.PDF]
