# Supplementary material for: Fine Mapping of a Novel Heading Date Gene, TaHdm605, in Hexaploid Wheat
Source: Front Plant Sci. 2018 Jul 18;9:1059. doi: 10.3389/fpls.2018.01059 (PMC6058285; doi:10.3389/fpls.2018.01059)
Supplement: FIGURE S2 — The expression profiles of the candidate genes on WheatEXP. [file Image_2.PDF]

gene1

Developmental timecourse in five tissues

Expression Data Graph

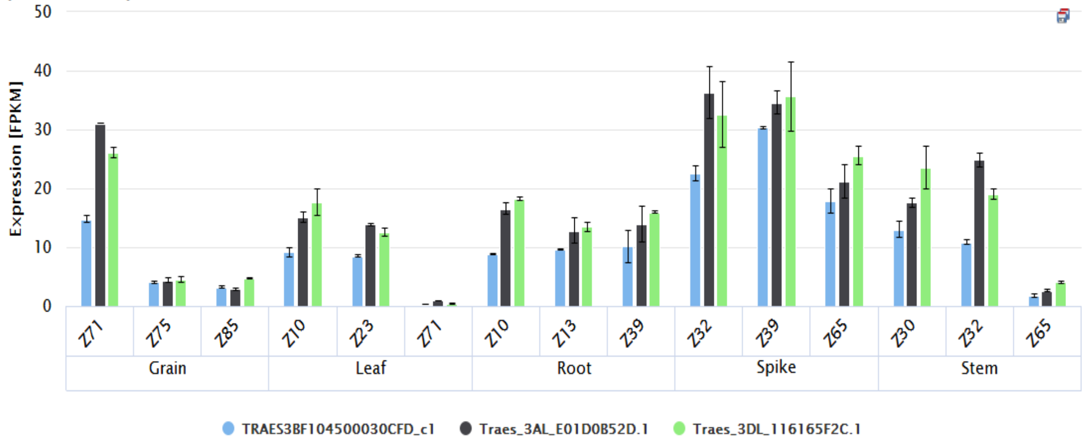

gene2

Developmental timecourse in five tissues

Expression Data Graph

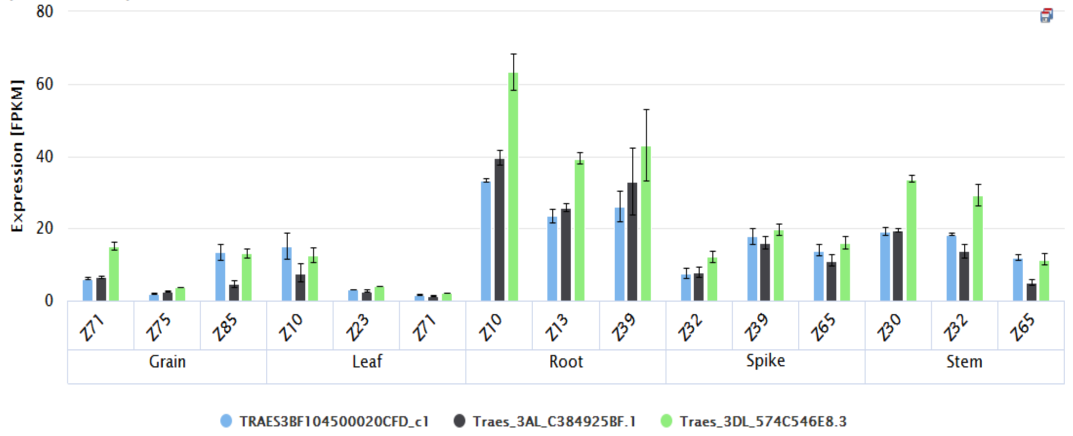

gene3

Developmental timecourse in five tissues

Expression Data Graph

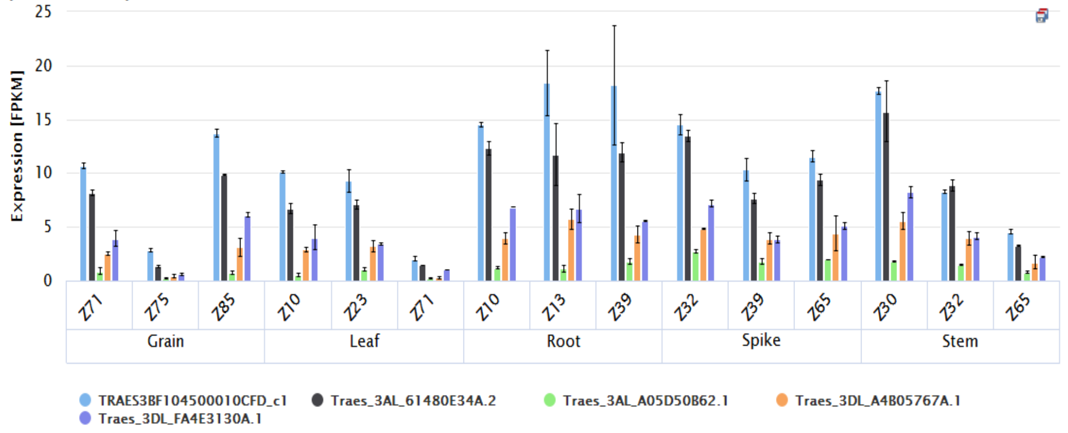

gene4 -----
gene5

Developmental timecourse in five tissues

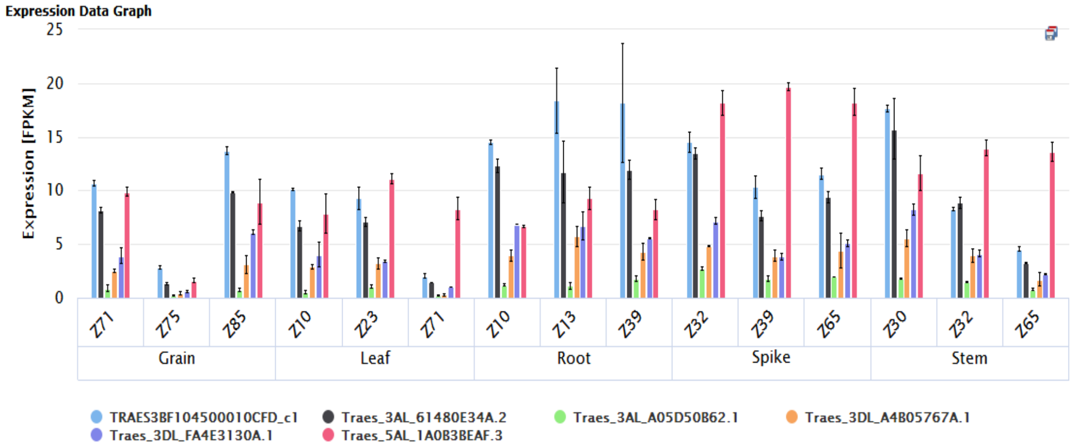

gene6

Developmental timecourse in five tissues

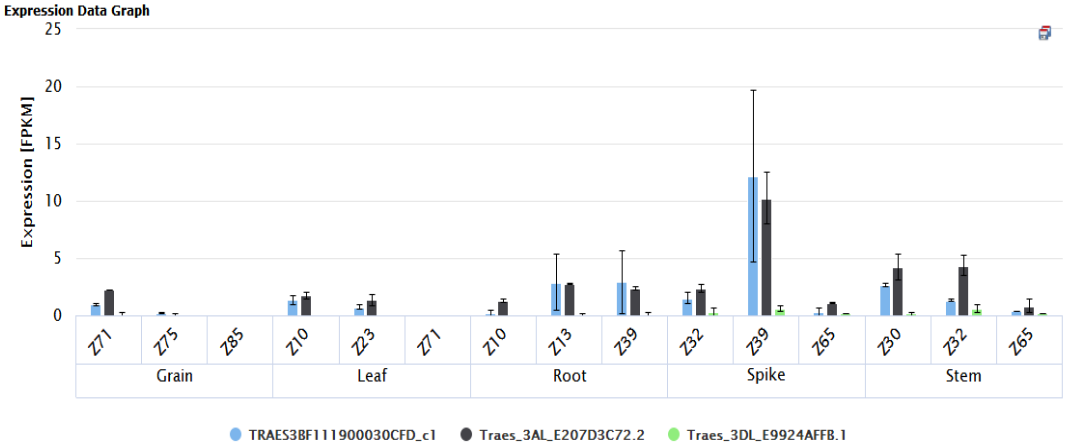

gene7 ----
gene8

Developmental timecourse in five tissues

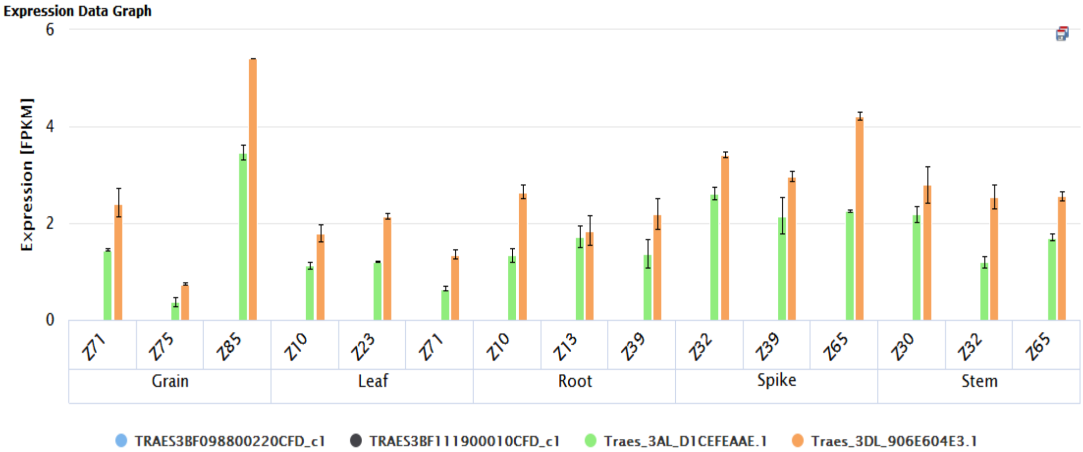

gene9

Developmental timecourse in five tissues

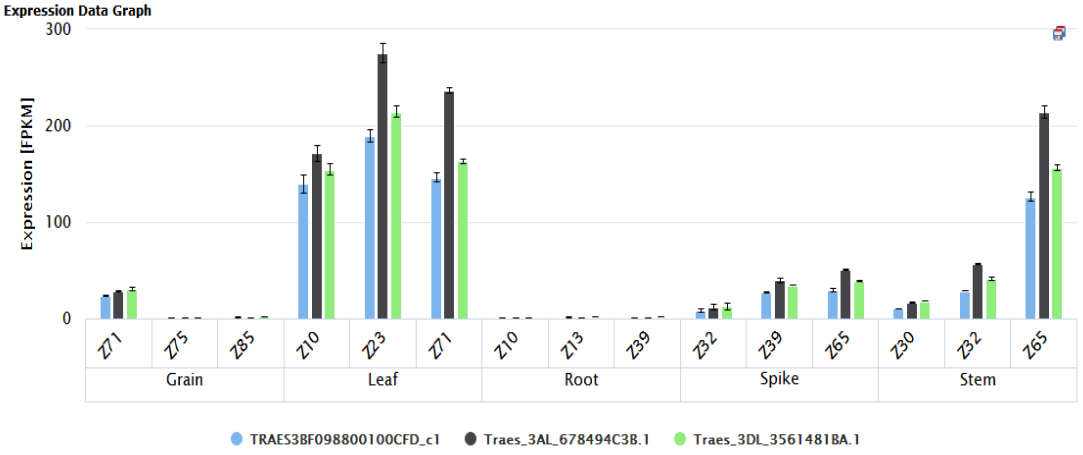

gene10

Developmental timecourse in five tissues

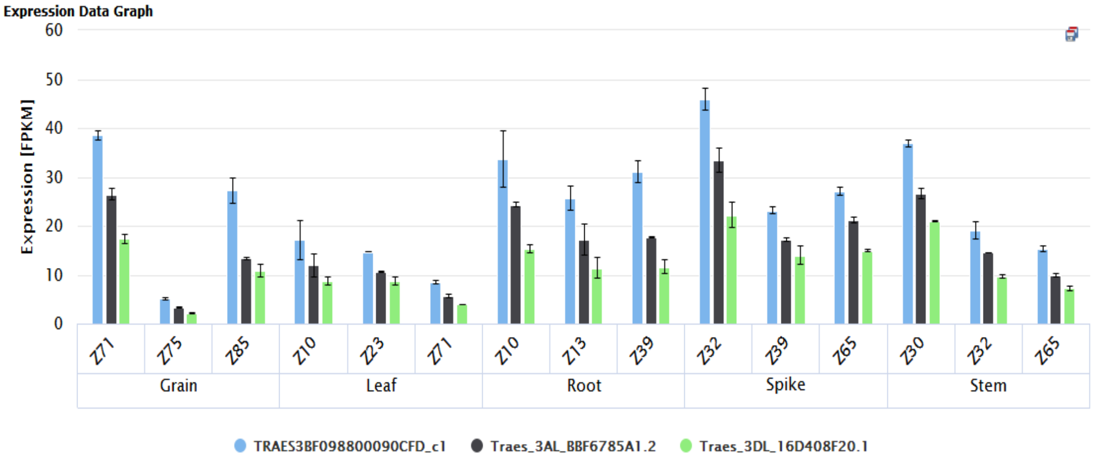

gene11 ----

gene12 ----

gene13 ----

gene14 ----

gene15 ----

gene16 ----

gene17

Developmental timecourse in five tissues

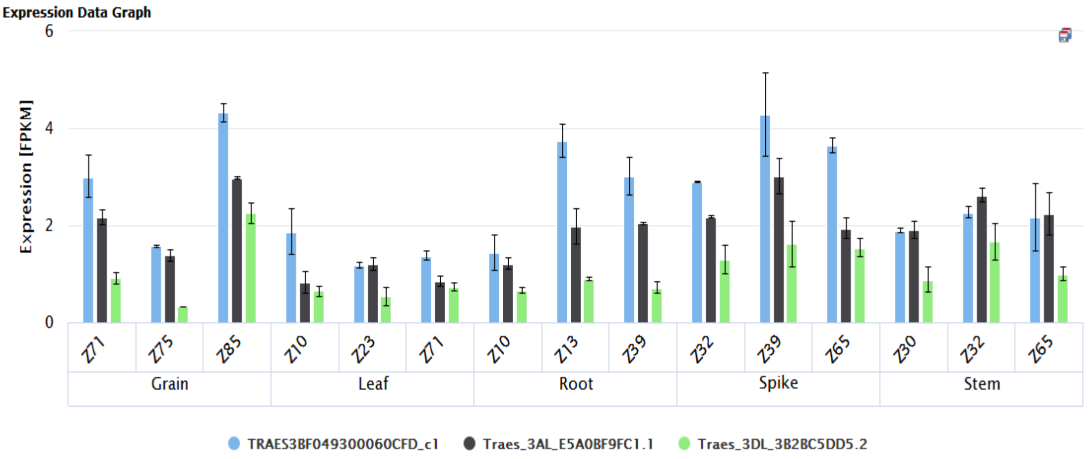

gene18

Developmental timecourse in five tissues

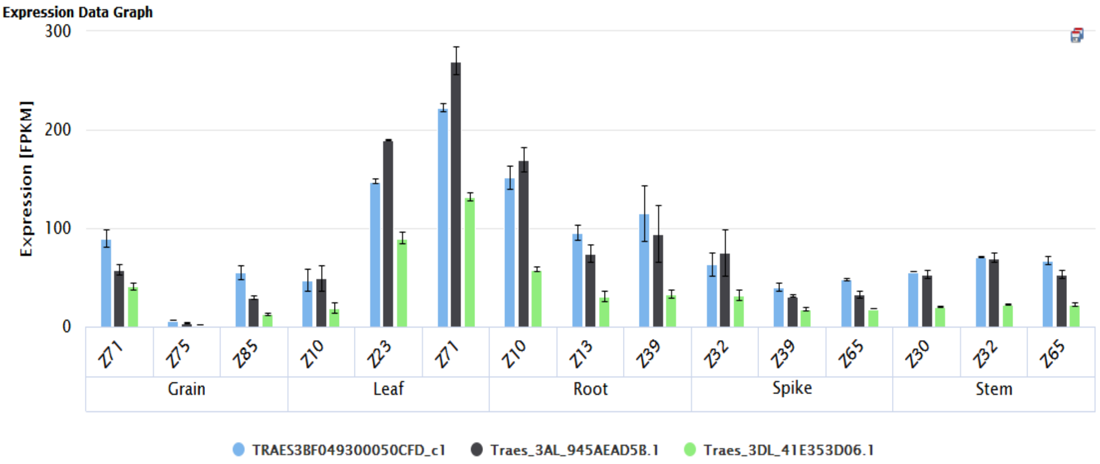

gene19

Developmental timecourse in five tissues

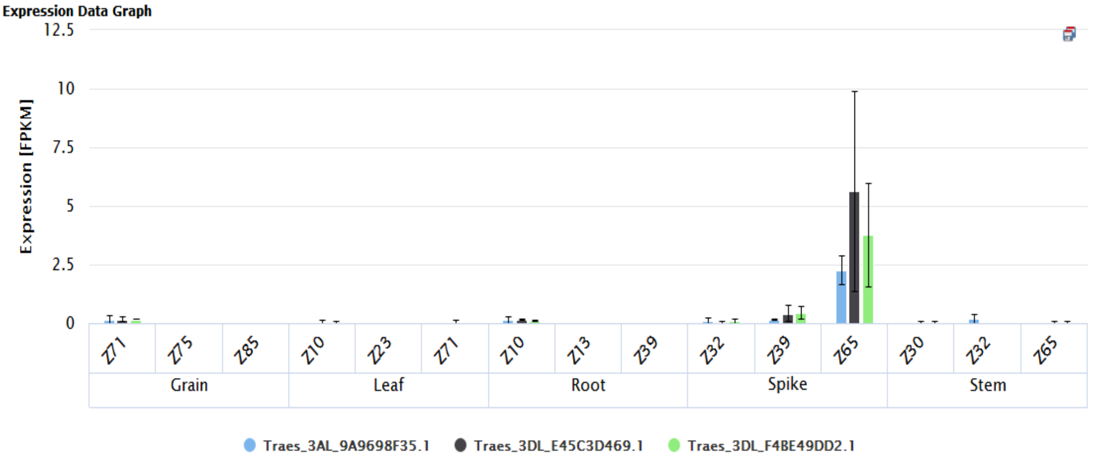

gene20

Developmental timecourse in five tissues

Expression Data Graph

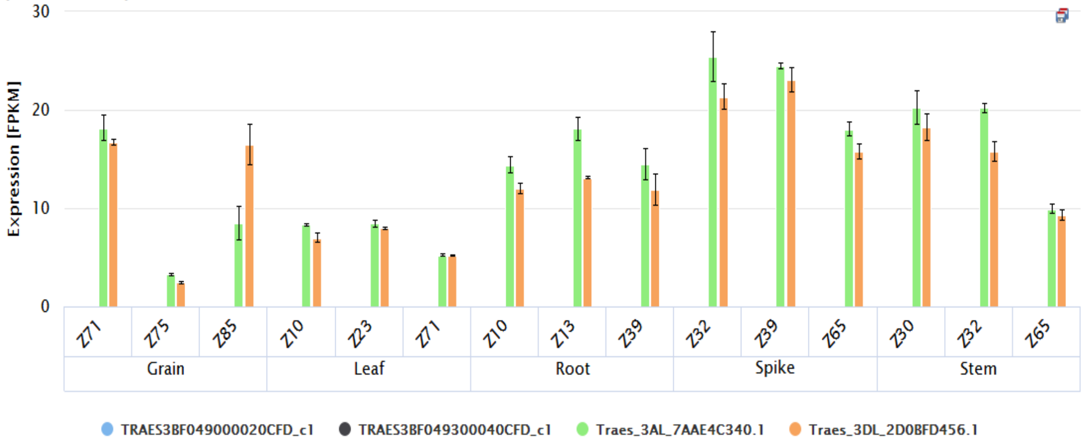

gene21

Developmental timecourse in five tissues

Expression Data Graph

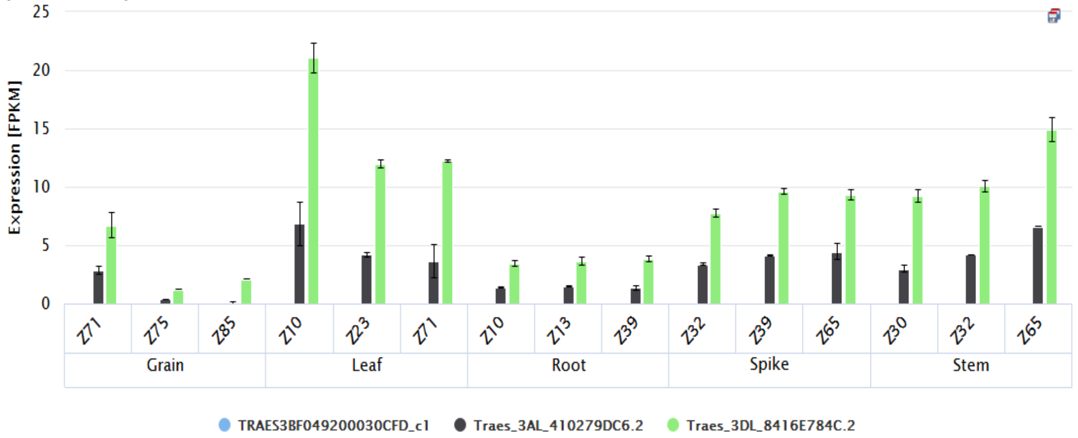

gene22

Developmental timecourse in five tissues

Expression Data Graph

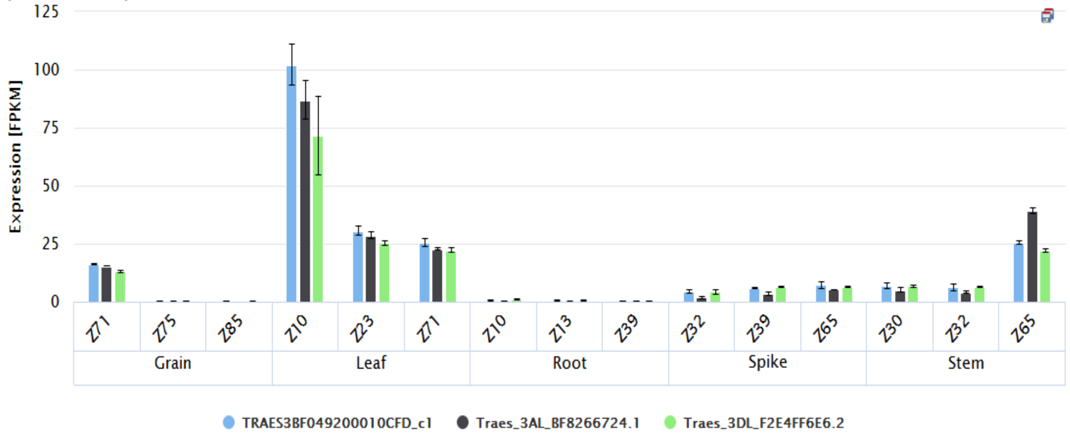

gene23

Developmental timecourse in five tissues

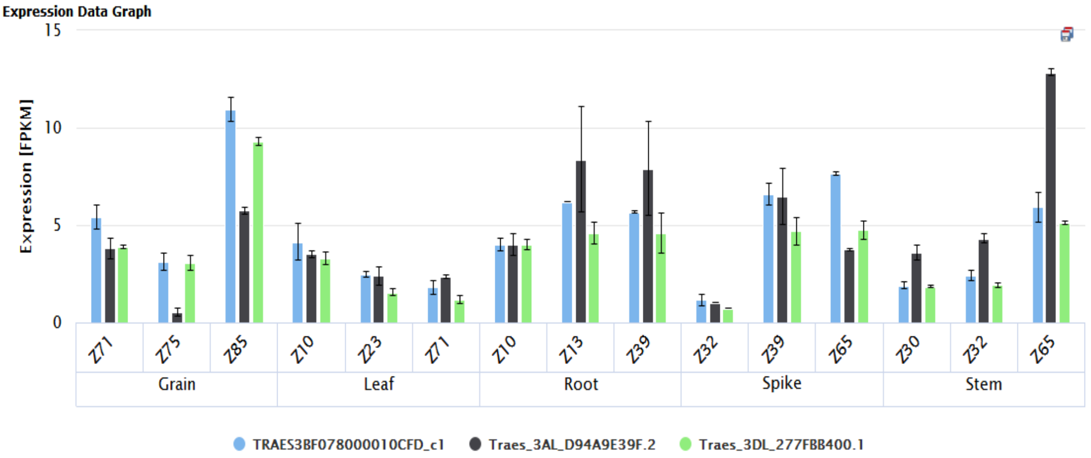

gene24 ---

gene25 ---

gene26 ---
