# Supplementary material for: Fine Mapping of a Novel Heading Date Gene, TaHdm605, in Hexaploid Wheat
Source: Front Plant Sci. 2018 Jul 18;9:1059. doi: 10.3389/fpls.2018.01059 (PMC6058285; doi:10.3389/fpls.2018.01059)
Supplement: TABLE S2 — The comparison of days to heading between YZ4110 and m605. [file Table_2.DOCX]

**Table S2** The comparison of days to heading between YZ4110 and *m605*

| accession | years | location | No.^#^ | days to heading |
| --- | --- | --- | --- | --- |
| YZ4110 | 2009 | Beijing | 20 | 218.9±0.55 |
| *m605* |  |  |  | 239.15±0.59** |
| YZ4110 | 2009 | Luoyang | 20 | 211.85±0.49 |
| *m605* |  |  |  | 226.05±0.39** |
| YZ4110 | 2010 | Beijing | 20 | 241±0.32 |
| *m605* |  |  |  | 250.3±0.80** |
| YZ4110 | 2011 | Beijing | 20 | 224.75±0.55 |
| *m605* |  |  |  | 237.2±0.70** |
| YZ4110 | 2011 | Xinxiang | 20 | 223.15±0.37 |
| *m605* |  |  |  | 232.2±0.62** |
| YZ4110 | 2012 | Beijing | 20 | 234.25±0.85 |
| *m605* |  |  |  | 245.15±0.67** |
| YZ4110 | 2013 | Beijing | 20 | 238.7±0.57 |
| *m605* |  |  |  | 246.1±0.31** |
| YZ4110 | 2014 | Beijing | 20 | 219.1±0.71 |
| *m605* |  |  |  | 239±0.92** |
| YZ4110 | 2015 | Beijing | 20 | 260.15±0.59 |
| *m605* |  |  |  | 265.85±0.88** |
| YZ4110 | 2016 | Beijing | 20 | 224.2±0.61 |
| *m605* |  |  |  | 235.05±0.76** |

^#^indicates the number of plants analyzed

*p<0.05; **p<0.01
