# Supplementary material for: Fine Mapping of a Novel Heading Date Gene, TaHdm605, in Hexaploid Wheat
Source: Front Plant Sci. 2018 Jul 18;9:1059. doi: 10.3389/fpls.2018.01059 (PMC6058285; doi:10.3389/fpls.2018.01059)
Supplement: TABLE S3 — The significance test of the interactions between TaHdm605 alleles, photoperiods, and vernalization treatments. [file Table_3.DOCX]

**Table S3** The significance test of the interactions between *TaHdm605* alleles, photoperiods, and vernalization treatments

| Source | DF | Type Ⅲ SS | Mean Square | F Value | Pr > F |
| --- | --- | --- | --- | --- | --- |
| *TaHdm605* alleles | 1 | 7068.80000 | 7068.80000 | 1663.25 | <.0001 |
| photoperiod | 1 | 1960.20000 | 1960.20000 | 461.22 | <.0001 |
| vernalization | 1 | 20672.45000 | 20672.45000 | 4864.11 | <.0001 |
| alleles * photoperiod | 1 | 1.80000 | 1.80000 | 0.42 | 0.5172 |
| alleles * vernalization | 1 | 2808.45000 | 2808.45000 | 660.81 | <.0001 |
