# Supplementary material for: Fine Mapping of a Novel Heading Date Gene, TaHdm605, in Hexaploid Wheat
Source: Front Plant Sci. 2018 Jul 18;9:1059. doi: 10.3389/fpls.2018.01059 (PMC6058285; doi:10.3389/fpls.2018.01059)
Supplement: TABLE S5 — Agronomic trait analysis based on the F2 population from the cross Chun47 × m605. [file Table_5.DOCX]

| trait | environment | 1,000-kernel weight | plant weight | grain number per plant | grain number per spike | spikelet number | spike length | effective tiller number | plant height |
| --- | --- | --- | --- | --- | --- | --- | --- | --- | --- |
| heading date | Beijing | -0.207** | -0.340** | -0.305** | -0.413** | -0.425** | -0.283** | -0.018 | -0.186** |
|  | Xinxiang | -0.541** | -0.408** | -0.524** | -0.567** | -0.380** | -0.301** | -0.147** | -0.472** |
| plant height | Beijing | 0.345** | 0.489** | 0.386** | 0.402** | 0.157** | 0.375** | 0.197** |  |
|  | Xinxiang | 0.541** | 0.353** | 0.399** | 0.412** | 0.211** | 0.267** | 0.227** |  |
| effective tillering | Beijing | -0.088* | 0.660** | 0.734** | 0.174** | 0.081* | 0.200** |  |  |
|  | Xinxiang | 0.111** | 0.167** | 0.277** | 0.180** | 0.079** | 0.135** |  |  |
| spike length | Beijing | 0.096** | 0.386** | 0.373** | 0.469** | 0.406** |  |  |  |
|  | Xinxiang | 0.199** | 0.215** | 0.261** | 0.336** | 0.211** |  |  |  |
| spikelet number | Beijing | 0.021 | 0.306** | 0.321** | 0.364** |  |  |  |  |
|  | Xinxiang | 0.181** | 0.166** | 0.305** | 0.381** |  |  |  |  |
| grain number per spike | Beijing | 0.203** | 0.502** | 0.470** |  |  |  |  |  |
|  | Xinxiang | 0.346** | 0.321** | 0.546** |  |  |  |  |  |
| grain number per plant | Beijing | -0.007 | 0.940** |  |  |  |  |  |  |
|  | Xinxiang | 0.313** | 0.541** |  |  |  |  |  |  |
| plant weight | Beijing | 0.270** |  |  |  |  |  |  |  |
|  | Xinxiang | 0.351** |  |  |  |  |  |  |  |
| 1,000- kernel weight | Beijing |  |  |  |  |  |  |  |  |
|  | Xinxiang |  |  |  |  |  |  |  |  |

**Table S5** Agronomic trait analysis based on the F_2_ population from the cross Chun47 × *m605*

**p*<0.05*; **p*<0.01
