# Supplementary material for: Fine Mapping of a Novel Heading Date Gene, TaHdm605, in Hexaploid Wheat
Source: Front Plant Sci. 2018 Jul 18;9:1059. doi: 10.3389/fpls.2018.01059 (PMC6058285; doi:10.3389/fpls.2018.01059)
Supplement: TABLE S6 — Newly developed polymorphic SSR markers tightly linked to the TaHdm605 locus. [file Table_6.DOCX]

**Table S6** New developed polymorphic SSR markers tightly linked to the *TaHdm605* locus

| markers | forward primers (5’-3’) | reverse primers (5’-3’) | product size (bp) | dominance |
| --- | --- | --- | --- | --- |
| D1 | ATGATCATGGATACGATTGAG | TCTGTGTTCTTTATTGGATGG | 157 | Co-dominant |
| M193 | CACAGGCCATATTTCTTTTATT | TGTGAGGTGGTTGTAAAAACT | 183 | Co-dominant |
| S59 | CAGTGGACACGCAGTACACA | ATGCATGTGTGTGTGCTCAA | 161 | Co-dominant |
| M195 | GGCGATTCACCCTACTTATAC | CCCAGTTCAAAAATATAGCAG | 135 | Co-dominant |
| C20 | CATCACAGGTGGAGGGATTT | CTTTCCCATCGCCGTGTTAT | 164 | Co-dominant |
| Q16 | TTCTTGAGAATGTCCTTGGTA | AGAATTTCGAGATGTCACTCA | 351 | Co-dominant |
| M18 | TGATGCAGGTATACGTTGTAA | TGTGCATGGTTCTTTTCTACT | 157 | Co-dominant |
| M11 | GAACCGATGTCTTTCAGAATA | ATGGCCGAAGTAGTATGTGTA | 148 | Co-dominant |
| M310 | GCAATGGTTATCTACATTGGA | ATACAGGTGAGAAAAACGACA | 142 | Co-dominant |
| M35 | TCATGAGCTAGAAACCAACTC | GGCTACATAGCAACAAGAGTG | 174 | Co-dominant |
| M27 | ATTACCATCAATTGCGTTATG | TGCGGAAGTGTTATCAGAATA | 135 | Co-dominant |
| Q26 | TGTAGTGACCATGGTTGAAGT | CTGTTGTTCCGATAGGTTATG | 145 | Co-dominant |
| M80 | AGAGCGCAGAAACAAAGGAA | GGGGGAAAGTCTTCTGCTTT | 166 | Co-dominant |
| S2 | GGATAACCTTGAGAAGGTAGG | ATTGGATGTCATGATCACTTG | 150 | Co-dominant |
